# Personal

**Published:** 1907-12

**Authors:** 


### PERSONAL.

DR. NELSON W. WILSON, late U.S.A., has been appointed a member of the literary committee of the Association of Military Surgeons of the United States.

DRS. WILLIAM L. AND FREDERICK J. PARMENTER, of Buffalo, have changed their residence from 931 Prospect Avenue to 616 Potomac Avenue, between Elmwood Avenue and Bidwell Parkway.

DR. F. PARK LEWIS, one of the distinguished ophthalmologists of Buffalo, was elected president of the Libera Club at its first session held this season November 22, 1907.

Dr. James J. Mooney, of Buffalo, has removed from 392 Seventh Street to 490 Delaware Avenue, between Virginia and Allen Streets. His practice is limited to diseases of the ear, nose, and throat.

---

Dr. Herman D. Andrews, of Buffalo, has established his offices at The Colonial, 399 Delaware Avenue, where he will continue the practice of his specialty—ophthalmology. Hours: 9 to 1. Telephones: Bell, Tupper 501; Frontier, 2375.

---

Dr Lewis S. McMurtry and Dr. J. Garland Sherill, of Louisville, have removed their offices from Masonic Temple to the Atherton Building, Suite 610, corner of Fourth Avenue and Chestnut Street. Hours: 11 to 1. Telephones: Cumberland Main 4100; Home 1700.

---

Dr. Floyd S. Crego, 503 Delaware Avenue, Buffalo,, announces change in office hours as follows: 9 to 10 A.M., 2 to 4 P.M. Evenings by appointment. Both phones. His specialty is nervous and mental diseases.

---

Dr. Thomas D. Strong, of Westfield, N. Y., celebrated his eighty-fifth birthday Friday, November 22, 1907. The medical profession of Chautauqua escorted him to Mayville, where a dinner was tendered him.

